# Process and Mechanism of Cutting Polyamide Films with an Ultraviolet Picosecond Laser

**DOI:** 10.3390/mi17070804

**Published:** 2026-06-30

**Authors:** Qin Xie, Tian Wang, Yan Zhou, Zeyue Gao, Jie Jiang, Congyi Wu, Bing Wei, Yu Huang

**Affiliations:** 1 School of Mechanical Science and Engineering, Huazhong University of Science and Technology, Wuhan 430074, China; qinxie@hust.edu.cn (Q.X.); zeyuegao@163.com (Z.G.); wucongyi@hust.edu.cn (C.W.); yuhuang_hust@hust.edu.cn (Y.H.); 2 Shanghai Institute of Space Power-Sources, Shanghai 200245, China; wangtian415@163.com (T.W.); 13817747686@139.com (Y.Z.); 3 School of Mechanical Engineering, Hubei University of Technology, Wuhan 430068, China; jiejiang_245@163.com; 4 Guangdong HUST Industrial Technology Research Institute, Dongguan 523808, China; 5 Guangdong Intelligent Robotics Institute, Dongguan 523808, China

**Keywords:** laser cutting, laser ablation, polyamide (PA), precision manufacturing

## Abstract

Polyamide (PA) films have been widely utilized in high-precision medical devices and aerospace components, while laser precision cutting technology has significantly broadened their application scope. Although ultraviolet (UV) picosecond lasers are effective for high-precision cutting of PA films, their cutting mechanism and the optimization method for the process remain to be elucidated. First, the mechanism of UV picosecond laser cutting of PA films was investigated through a simulation of the thermal degradation process and analysis of the solid/gas byproduct composition. The results indicate that the photochemical reaction primarily dominates the process, with the photothermal effect contributing synergistically. Second, a cutting quality evaluation framework was established, with the kerf width and heat-affected zone (HAZ) width as its primary metrics, followed by an orthogonal experiment. The experimental results revealed the influence of process parameters on the cutting quality, and it was determined that an optimal process parameter combination exists, identified as 80 mm/s, 1.67 W, and three times (cutting speed, laser power, repetition number of cutting). Under this optimal configuration, narrow kerf (23.6 ± 2.7 μm) and HAZ (28.4 ± 3.3 μm) were achieved.

## 1. Introduction

Polyamide (PA) exhibits a number of advantageous properties, including excellent mechanical strength [1,2], wear resistance [3], chemical stability [4], heat resistance [5], and self-lubricating properties [6]. Consequently, it is widely applicable in packaging [7,8], textile industries [9], medical devices [10], electronic equipment [11], automotive components [12], and the aerospace industry [13]. Given the expanding range of PA films, there is an increasing demand for cutting precision and quality, driving the need for advanced processing techniques.

Conventional machining processes such as turning and milling are widely employed for large-scale processing of PA plates and bars, yet the cutting forces generated by tool contact lead to defects such as deformation, burrs, and cracking in the film, making it difficult to process complex contours and delicate patterns [14,15]. Ultrasonic cutting relies on high-frequency tool vibration for material removal, with reduced contact pressure and no material fracture, but it suffers from high equipment costs and rapid tool wear [16]. Wire electrical discharge machining exhibits negligible mechanical stress and enables precision cutting of complex shapes, but it is unsuitable for insulating materials like PA films [17]. Water jet cutting offers the advantages of no thermal deformation and good edge quality, but water jet diffusion and deflection constrain its cutting precision [18]. These limitations present significant technical challenges for high-precision and high-quality cutting of PA films, necessitating the development of alternative processing methods.

Laser cutting offers significant advantages, including high efficiency [19], high precision [20], good cut quality [21], a flexible cutting path [22], and material adaptability [23,24,25,26,27,28]. Compared with CO2 lasers [29] and fiber lasers [30], pulsed lasers demonstrates higher peak power and narrower pulse width, enhancing cutting precision and quality. UV lasers are particularly suitable for polymer processing due to their high absorption characteristics [31]. Picosecond laser cutting combines high precision, high efficiency, moderate cost and stable performance, making it ideal for industrial-scale production [32]. Liu et al. [33] fabricated high-quality microholes in polyimide films using a UV picosecond laser, and Götze et al. [34] demonstrated the feasibility of processing polyamide electrospun nanofibers by UV picosecond laser irradiation. These studies highlight the great potential of UV ultrashort-pulse lasers for precision polymer processing. However, continuous PA films differ substantially from nanofiber structures in terms of morphology, heat transfer behavior, and laser–material interaction. Despite the extensive application of PA films in aerospace components and precision devices, systematic studies on UV picosecond laser cutting of PA films remain scarce. In particular, the material removal mechanism, byproduct evolution, and the relationships between processing parameters and cutting quality have not yet been clarified.

In this study, a UV picosecond laser was selected for precision cutting of PA films. Analysis of byproducts and preliminary process experiments were employed to establish the laser cutting mechanism, which is dominated by the photochemical reaction and synergized by the photothermal effect. In addition, quality evaluation criteria were developed, and orthogonal experiments were conducted to systematically investigate the influence of parameters on cutting quality. The optimal parameter combination (80 mm/s, 1.67 W, three times) achieves balanced cutting quality and efficiency, obtaining a kerf width of 23.6 ± 2.7 μm and a HAZ width of 28.4 ± 3.3 μm, with complete through-cutting and a well-defined kerf. The results provide both mechanistic understanding and practical guidance for precision machining of PA films, particularly for aerospace applications.

## 2. Experiment

### 2.1. Materials and Laser Cutting System

Polyamide (PA) films with a thickness of 100 μm were purchased from Yuxin Plastic Materials (Dongguan, Guangdong, China). Ethanol solution (analytical grade, 99.7%, 500 mL) and deionized water were obtained from Sinopharm Chemical Reagent Co., Ltd. (Shanghai, China). To eliminate potential interference from surface contaminants such as oil and dust during the laser cutting process, the PA films were ultrasonically cleaned sequentially with ethanol and deionized water for 10 min each, followed by air drying at room temperature. After laser cutting, the samples underwent the same cleaning and drying procedures to ensure the reliability of subsequent characterization.

**Figure 1:**
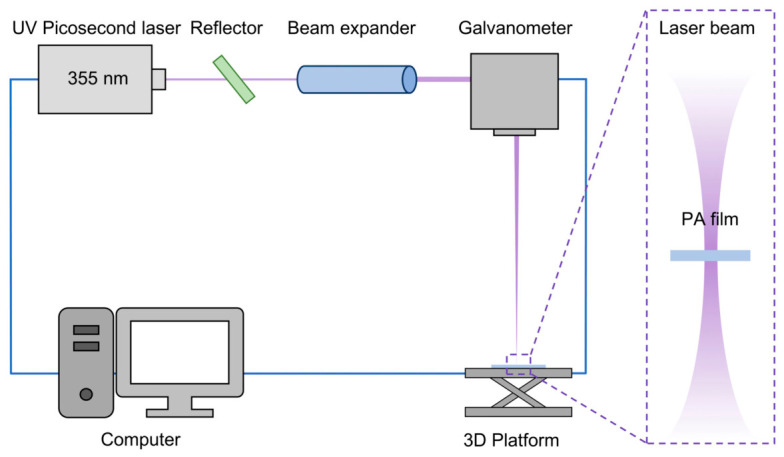
Schematic diagram of laser cutting system.

In this study, a UV picosecond laser processing system was employed for the experimental investigation of the fabrication process. The system consists of optical and control modules, as illustrated in Figure 1 The optical module includes a UV picosecond laser, a beam expander, reflectors, and a three-dimensional scanning galvanometer. The pulsed laser beam is emitted from the laser source and directed through a reflector. Then, it passes through the beam expander to enhance its collimation and directivity. Subsequently, the beam enters the scanning galvanometer, finally being focused onto the sample surface by the focusing lens (d > 11.8 μm). The control module comprises an industrial control computer and a motion platform. The industrial control computer is equipped with a control card and specialized software, enabling precise regulation of laser processing parameters and real-time control of the deflection angles to adjust the two-dimensional scanning path of the laser beam. The sample is mounted on the motion platform, which allows movement in the X, Y, and Z directions, ensuring that the processing area of the PA film remains within the focal plane of the laser. The main process parameters for the laser cutting system are listed in Table 1.

### 2.2. Design of Experiment

**Figure 2:**
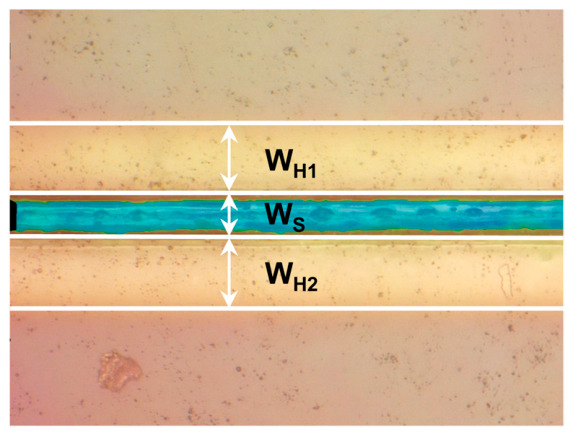
Diagram of the PA film laser cutting quality evaluation.

A multi-parameter quantitative evaluation method was established based on the typical laser-cut morphological features to assess the laser-cutting quality of PA films. As illustrated in Figure 2, a kerf was formed along the scanning path on the PA film surface after laser processing, with its maximum cross-sectional width defined as the kerf width (W_S_). The HAZ was formed in the edge regions adjacent to the kerf due to laser–material interactions, exhibiting significant differences from the base material. The upper and lower HAZ exhibit an asymmetric distribution due to the combined effects of material properties, heat conduction, and processing conditions. To establish a unified quantitative evaluation index, the width of the upper (W_H1_) and lower HAZ (W_H2_) were measured respectively, and the maximum value was adopted as the characteristic parameter for HAZ evaluation, calculated by the following formula: W_H_ = MAX (W_H1_, W_H2_).

Preliminary process experiments identified three critical parameters influencing cutting quality: laser power, cutting speed, and repetition number of cutting. Among these, the laser power and the cutting speed primarily affect the width of the kerf and HAZ, while the repetition number of cutting mainly influences the kerf morphology and cutting depth. Preliminary experimental results determined the optimal range of parameters. With a laser power range from 1 to 3 W and a cutting speed range from 20 to 100 mm/s, satisfactory cutting quality without significant ablation defects can be obtained. The laser power regulation was implemented through percentage adjustment of total energy output, showing slight deviations between its actual output power and the preset value, with actual power ranging from 1.15 to 3.18 W. A two-stage experimental design strategy was adopted following the principle of controlled variables. Initially, a 2-factor, 5-level orthogonal experiment was designed to investigate the effects of the laser power and the cutting speed on cutting quality, with factor levels given in Table 2. The repetition number of cutting was fixed at 1 in this stage to eliminate its interference. Subsequently, cutting speed and laser power were maintained at optimal levels. The repetition number of cutting was increased to ensure that the PA film was wholly cut through, and its effect on the cutting quality was explored.

### 2.3. Characterization

The morphological features, byproducts, and thermal behavior of the PA film were synergistically analyzed using multi-scale characterization techniques. Initial surface morphology observation and precise dimensional measurements of the kerf and heat-affected zone were conducted with a Super-Depth-of-Field Microscope (SDM, VHX-7000, KEYENCE, Osaka, Japan). An environmental scanning electron microscope (ESEM, Quanta 200, Hillsboro, OR, USA) was employed to obtain high-resolution images of the sample surface micromorphology, and an energy-dispersive spectrometer (EDS, Quanta 200) was utilized to analyze the elemental species and content of the material microzone constituents around the kerf. Meanwhile, Raman spectroscopy (785 nm laser, HORIBA Jobin Yvon, Palaiseau, France) was employed to acquire Raman spectra around the kerf, characterizing its molecular structure in typical areas for further analysis of the solid byproducts. A rapid thermal cracking experiment (25~1000 °C) was carried out on the PA film to simulate the laser ablation process, with the gaseous byproducts analyzed qualitatively and quantitatively by gas chromatography–mass spectrometry (GC-MS, 7890A/5975C, Agilent, Santa Clara, CA, USA). Thermogravimetry–Fourier transform infrared spectroscopy (TG-FTIR, PerkinElmer, Waltham, MA, USA, 20 °C/min, 25~800 °C, N_2_) was employed to simultaneously monitor the thermal weight loss process of the material and the evolution of the gas composition.

## 3. Results and Discussion

### 3.1. Analysis of Byproducts

Laser cutting of polymer materials is primarily governed by two fundamental mechanisms: the photochemical reaction and the photothermal effect [35,36]. Preliminary experimental results have demonstrated a narrow HAZ along the kerf edges without prominent thermal damage characteristics such as melting, cracking, or burring, which significantly differs from typical photothermal-dominated cutting features. Consequently, it can be preliminarily inferred that photochemical reactions play a dominant role during UV picosecond laser cutting of the PA film, while photothermal effects exhibit synergistic contributions. To further elucidate the interaction mechanism between the ultraviolet picosecond lasers and the PA film, a systematic analysis of the dynamic change in the material composition and the generation of byproducts during the laser cutting process is necessary.

**Figure 3:**
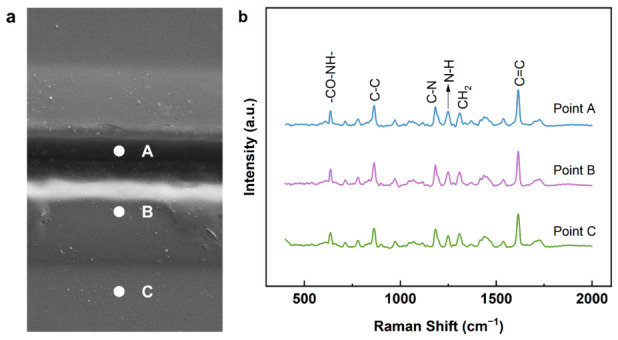
(**a**) ESEM image showing the selected Raman measurement locations; (**b**) Raman diagram at different positions around the kerf.

Raman spectroscopy and EDS were first employed to analyze solid byproducts in and around the kerf to clarify the evolution of material composition during laser processing. As shown in Figure 3a, three characteristic positions (points A, B, and C) were selected from the inner to the outer surface of the uncleaned PA film after cutting, where point A represents the inner wall of the kerf, point B corresponds to the HAZ and point C indicates the unprocessed region. The Raman spectra test results are depicted in Figure 3b, showing no significant differences among the spectra obtained from the three points. Notably, no distinct carbon characteristic peaks were observed at 1332 cm^−1^ (D band) or 1580 cm^−1^ (G band) [37], with all detected peaks corresponding to the PA material. Therefore, UV picosecond laser cutting of PA did not induce carbonization from excessive ablation. Consequently, no apparent solid byproducts remained in the kerf or surrounding HAZ under these processing conditions. As presented in Table 3, the EDS analysis results exhibited negligible variation (2.2%) in carbon and oxygen atomic percentage among the three points, demonstrating that the material composition in the processed regions remained consistent with the original PA film surface. These results further verify that the laser–PA material interaction did not result in significant carbonization or solid residues.

**Figure 4:**
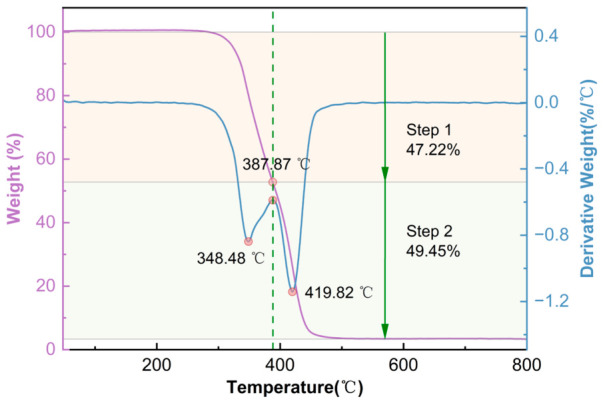
TG and DTG curves of the PA film.

TG analysis, which reflects the weight variation pattern of the PA film under temperature elevation, was conducted to analyze the thermal reaction behavior of the PA film during laser cutting. Figure 4 presents the TG curve and derivative thermogravimetry (DTG) curve of the PA film under a heating rate of 20 °C/min in a nitrogen atmosphere. The TG curve reveals that the PA film is in a stable reaction plateau below 280 °C, without significant weight loss. As the temperature rises, the film enters the weight loss stage. Above 500 °C, the TG curve stabilized, with no noticeable weight changes with further temperature increases. The DTG curve, reflecting the rate of weight loss, was further analyzed to characterize the weight loss process. Two distinct weight loss rate peaks are observed in the DTG curve, indicating that the thermal weight loss of the PA film can be divided into two stages. In the first stage (280~387.87 °C), the PA film underwent primary thermal decomposition, exhibiting a weight loss percentage of 47.22%. The weight loss rate progressively increases with temperature, reaching its first peak at 348.48 °C. Subsequently, the weight loss rate decreases until an inflection point appears at 387.87 °C, marking the completion of the first stage. During the second stage (387.87~500 °C), the weight loss rate exhibits an initial increase, followed by a subsequent decrease, and a distinct peak occurs at 419.82 °C. As the temperature reaches 500 °C, the DTG curve approaches 0, indicating near completion of the thermal decomposition process. The cumulative weight loss after both stages reaches 96.67% of the original weight. The TG and DTG curves at different temperature stages were compared to analyze the stage-specific characteristics of the heat weight loss behavior. The lower onset temperature of the first stage suggests preferential decomposition of labile functional groups or thermally unstable structural components in the PA film. The second stage, occurring at higher temperatures, indicates that the components undergoing thermal weight loss in this stage are more thermally stable, requiring more severe temperature conditions. Although both stages exhibited a comparable weight loss percentage (47.22% vs. 49.45%), the significantly higher weight loss rate observed in the second stage demonstrates more intense decomposition reactions. It should be noted that discrepancies exist between the test environment (atmosphere and heating rate) of TG and the actual laser-cutting process conditions. Therefore, the results of TG cannot completely and accurately reflect the weight loss behavior of the PA film during actual processing.

**Figure 5:**
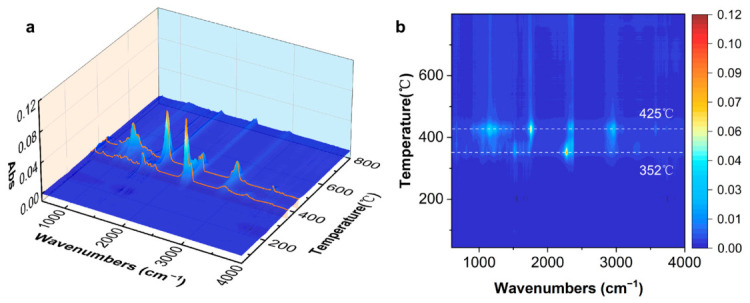
Infrared spectrogram of evolved gases in the PA film heating process. (**a**) Three-dimensional spectrogram; (**b**) Two-dimensional projection of the spectrogram.

The TG-FTIR technique was employed to analyze the composition of gaseous byproducts evolved during thermal decomposition, and the infrared spectra of the PA film (25~800 °C) are depicted in Figure 5. No distinct infrared absorption peaks are observed below 300 °C. With increasing temperature, weak peaks emerged and intensified progressively, reaching local maxima at 352 °C and 425 °C. Subsequently, the intensity of the vibrational peaks weakened again. These two peaks are located in the first and second stages of weight loss, which are close to the temperature points corresponding to the extremes of the weight loss rate, confirming the two-step thermal decomposition of PA films. Additionally, the infrared spectra at the characteristic temperature points corresponding to the most substantial vibrational peaks were analyzed to investigate the composition of the weight loss products in the two stages.

**Figure 6:**
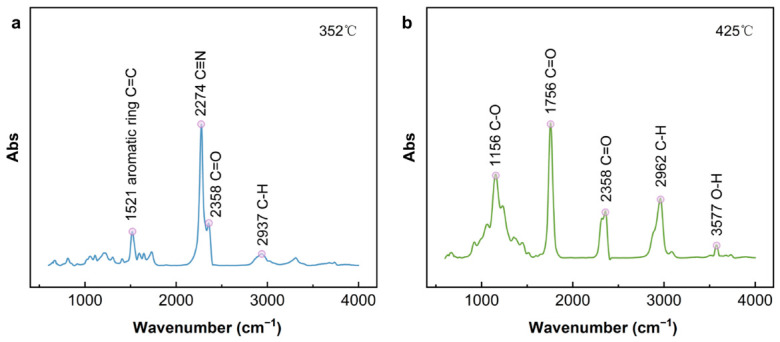
Infrared spectrogram at typical temperatures for each step. (**a**) Infrared spectrogram at 352 °C during the first stage; (**b**) Infrared spectrogram at 425 °C during the second stage.

The infrared spectrum at 352 °C during the first stage is depicted in Figure 6a, exhibiting four distinct infrared vibrational peaks at 1521 cm^−1^, 2274 cm^−1^, 2358 cm^−1^, and 2937 cm^−1^. The peak at 1521 cm^−1^ corresponds to C=C stretching vibrations in aromatic rings, indicating structural rearrangement or aromatization processes under thermal conditions. The peak at 2274 cm^−1^ is associated with C≡N stretching vibrations of cyano groups, suggesting bond breakage and rearrangement of amide linkages (-CONH-). The peak at 2358 cm^−1^ originates from asymmetric stretching of C=O bonds in CO_2_. This observation indicates that carbonyl groups within the molecular chain, such as the C=O of the amide bond, undergo further oxidation or pyrolysis, resulting in a subsequent release of CO_2_. The peak at 2937 cm^−1^ is attributed to C-H stretching vibrations in aliphatic chains, which is related to the structure of hydrocarbon groups, such as the methylene chain (-CH_2_-) of PA.

The infrared spectrum at 425 °C during the second stage is depicted in Figure 6b, exhibiting five distinct infrared vibrational peaks at 1156 cm^−1^, 1756 cm^−1^, 2358 cm^−1^, 2962 cm^−1^ and 3577 cm^−1^. The peak at 2358 cm^−1^ is related to stretching vibrations of C=O bonds in CO_2_, indicating the release of CO_2_ gas in this stage. At this stage, the absorption peak corresponding to aliphatic C-H stretching vibrations shifted to 2962 cm^−1^. With increasing temperature, the characteristic peak at 1512 cm^−1^ corresponding to aromatic rings disappeared, while new peaks emerged at 1156 cm^−1^, 1756 cm^−1^ and 3577 cm^−1^. The peak at 1156 cm^−1^ is attributed to C-O bonds, potentially originating from additives or ester compounds formed during degradation, while the vibration peak at 1756 cm^−1^ corresponds to the C=O stretching vibration, commonly observed in ester or ketone byproducts. The O-H vibration peak of free hydroxyl groups at 3577 cm^−1^ corresponds to hydroxyl-containing compounds such as alcohols generated during PA pyrolysis. Compared with the infrared spectrum results at 352 °C, the composition of byproducts becomes more complex at higher temperatures.

**Figure 7:**
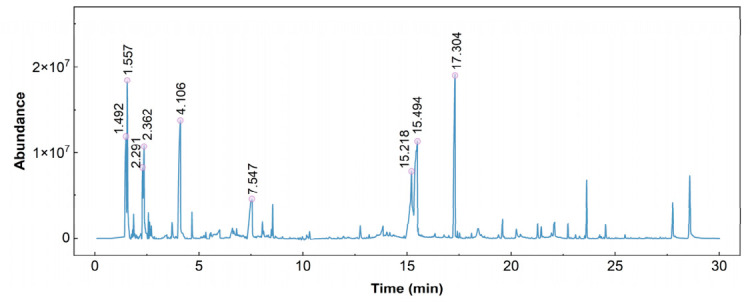
Ion current of evolved gases.

To provide complementary information regarding the thermal decomposition behavior of PA films, rapid pyrolysis experiments were performed, and the evolved gases were analyzed by GC-MS. The results are presented in Figure 7 and Table 4. N-methylmaleimide was the dominant product (12.18%), indicating that the amide bond of the main chain of PA material was broken through the cyclization reaction to generate maleimide derivatives. The double peaks of N-methylmaleimide reflect its isomer formation or differences in reaction kinetics at different stages. Additionally, cyclopentanone (7.49%) and adipic acid, dicyclobutyl ester (7.06%) were generated in significant quantities, suggesting the scission of aliphatic segments and esterification side reactions of PA. The thermal degradation of the methylene chains produced low-molecular-weight hydrocarbons such as propane (4.18%) and 1,3-butadiene (5.95%). Furthermore, the thermal cracking process was accompanied by the release of trace amounts of tetrahydrofuran (1.83%), 3-buten-1-ol (3.85%), and 1,4-butanediol (1.29%). In summary, the thermal pyrolysis of PA involves not only the cleavage of amide bonds but also complex secondary reactions such as cyclization, oxidation, and esterification. Notably, some gaseous products pose varying degrees of health hazards. N-methylmaleimide, as the predominant product, exhibits toxicity and may irritate the respiratory tract, skin, and eyes, with potential chronic inflammation upon prolonged exposure. 1,3-Butadiene is a known carcinogen, and tetrahydrofuran affects the central nervous system. It is imperative to implement comprehensive protective measures such as respirators, safety goggles, and gloves during the cutting process of PA films. Workplace ventilation systems should also be optimized by installing local and general ventilation equipment.

Based on the analysis of solid residues and thermal decomposition products, together with the fundamental principles of laser–material interaction, a possible material removal mechanism during UV picosecond laser cutting of PA films was discussed. The photon energy of the 355 nm laser is approximately 3.49 eV, which is comparable to the bond dissociation energy of C–N bonds (~3.16 eV) in polyamide. Moreover, PA exhibits appreciable absorption in the 340–400 nm spectral region [38]. Therefore, direct bond scission induced by photon absorption is possible, promoting photochemical decomposition and the formation of volatile products. Meanwhile, the photothermal effect plays a synergistic role. Despite suppressing thermal diffusion due to the ultrashort pulse duration, the instantaneous deposition of high-density laser energy induces localized high temperatures on the PA film surface, resulting in material melting or vaporization. The material is driven out of the cutting path by volatile products, forming a kerf, while thermal diffusion alters the properties of surrounding materials, contributing to the HAZ. These observations indicate that photochemical and photothermal effects jointly contribute to material removal, with photochemical bond scission playing a predominant role. Therefore, to achieve high-precision and high-quality cutting of the PA film, it is essential to optimize laser processing parameters to modulate the photochemical reaction and photothermal effect.

### 3.2. Analysis of Cutting Quality

**Figure 8:**
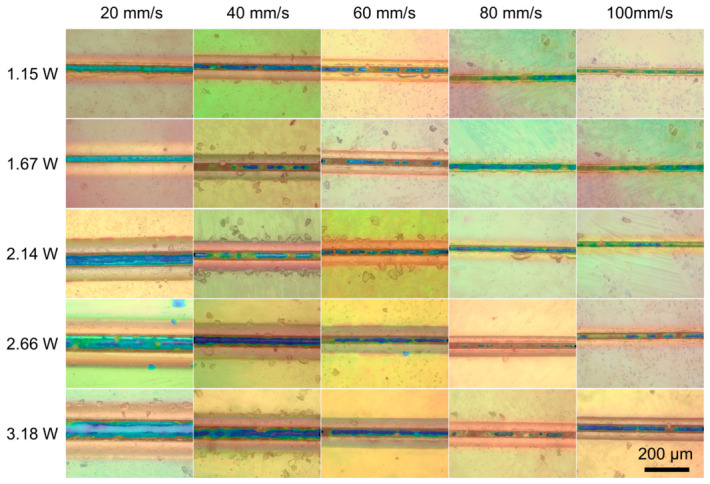
Typical SDM image of each sample.

A two-factor, five-level orthogonal experiment was employed to systematically investigate the influence of the laser power and the cutting speed on the cutting quality of the PA film. The kerf width (W_S_) and HAZ width (W_H_) were selected as the evaluation indexes, and five sets of sample data were collected under each combination of process parameters. Outliers were identified and removed using Grubbs’ test (α = 0.05), and the arithmetic mean of the remaining data in each dataset was used as the representative value. Typical SDM images are presented in Figure 8, while the averaged W_S_ and W_H_ values are summarized in Table 5. The experimental results demonstrate that when the laser power is constant, W_S_ and W_H_ initially decrease and then stabilize with the increased cutting speed. Conversely, when the cutting speed remains fixed, W_S_ and W_H_ positively correlate with the laser power, showing an initial reduction followed by stabilization as the power decreases. These observations can be explained by the fundamental principles of cutting. An increase in cutting speed decreases the laser energy deposition per unit time, and a decrease in laser power directly reduces the energy input. Increased cutting speed and decreased laser power effectively suppress thermal diffusion and heat accumulation effects, reducing W_S_ and W_H_. However, due to the nonlinear relationship between energy input and heat conduction, the material interaction tends to approach a critical threshold, causing the decreasing trends of W_S_ and W_H_ to stabilize gradually. Notably, excessively low energy input compromises material removal efficiency and even prevents the formation of the continuous kerf. Through comprehensive evaluation of cutting quality and processing efficiency, the best parameter combination within the investigated power–speed parameter space was identified as a laser power of 1.67 W and a cutting speed of 80 mm/s (Table 5, NO. 9). Under these conditions, the measured kerf width is 19.66 μm, while the HAZ width is 28.20 μm.

To further elucidate the influence mechanism of the laser power and the cutting speed on cutting quality (W_S_ and W_H_), the significance of the effects of different controllable factors on the dependent variables was quantitatively assessed by ANOVA. As presented in Table 6, the ANOVA results for W_S_ demonstrate that laser power, cutting speed, and their interaction exhibit statistically significant effects (*p* < 0.001). Notably, the cutting speed dominates with a contribution rate of 64.44%, substantially higher than that of laser power (25.66%) and their interaction effect (9.38%). Similarly, the ANOVA results for W_H_, listed in Table 7, reveal significant influences (*p* < 0.001) from these two parameters and their interaction. The cutting speed shows superior influence with a contribution rate of 68.20%, markedly exceeding the contribution rate of laser power (25.67%) and interaction effect (5.54%). In summary, the effects of both process parameters on cut quality were statistically significant. Appropriate selection of the laser power and the cutting speed contributes to cutting quality improvement, with the cutting speed demonstrating more pronounced regulatory effects on W_S_ and W_H_.

**Figure 9:**
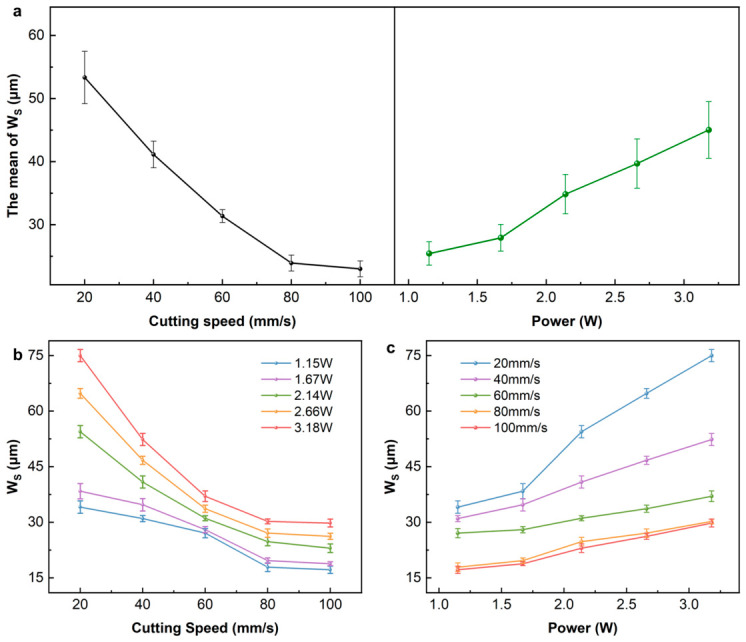
(**a**) Main effect diagram and (**b**,**c**) interaction effect diagram based on WS.

ANOVA was used to evaluate the influence of different variation sources by quantifying their contributions to total variation, which can be subdivided into main effect analysis and interaction effect analysis. The main effect analysis quantifies the independent influence of individual process parameters on the target metrics. As shown in Figure 9a, when the cutting speed increases from 20 mm/s to 100 mm/s, the mean of W_S_ decreases continually, indicating a significant negative correlation. Notably, the rate of reduction decreases in the high-speed range. Conversely, decreasing laser power from 3.18 W to 1.15 W leads to a consistent reduction in the mean of W_H_, demonstrating a positive correlation, with the rate of change becoming less pronounced in the low-power range. Interaction analysis reveals the synergistic effects between process parameters. Unparallel trend lines indicate the presence of interactions, and line crossings or inversions suggest stronger interactive influences. The interaction analysis results are shown in Figure 9b,c, demonstrating that across all power levels (1.15~3.18 W), the trends between the curves remain consistent, with higher cutting speeds decreasing W_S_. However, the slope is steeper at higher power levels, indicating that speed exerts a more pronounced regulatory effect on W_S_ under higher power conditions. Similarly, an increase in laser power at varying cutting speeds (20~100 mm/s) increases W_S_. Nevertheless, the interaction effect also leads to a difference in the slopes of the curves, with the increase in W_S_ due to the increase in laser power being more pronounced in the low-speed section (20~40 mm/s).

**Figure 10:**
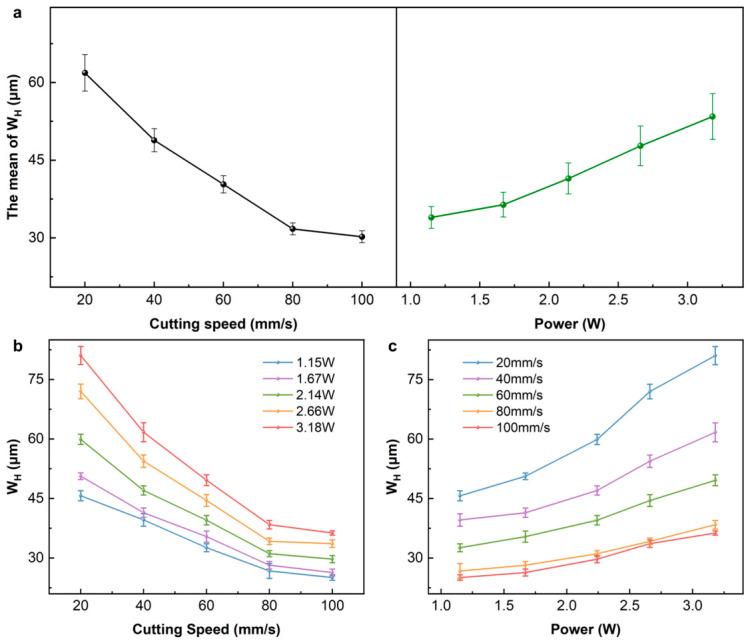
(**a**) Main effect diagram and (**b**,**c**) Interaction effect diagram based on WH.

The main effect analysis and the interaction effect analysis were performed with W_H_ as the evaluation index. As shown in Figure 10a, the main effect analysis demonstrates that increasing cutting speed from 20 mm/s to 100 mm/s leads to a continuous decrease in the mean value of W_H_, exhibiting a nonlinear negative correlation with diminishing reduction rate at higher speed. The interaction analysis result in Figure 10b indicates that decreasing cutting speed decreases WH at varying power levels, with the effect being more significant in the high-power range. Figure 10c illustrates the relationship between W_H_ and the laser power at different cutting speed levels, showing a markedly steeper slope in the low-speed range (20~40 mm/s), where increasing power causes a more substantial rise of W_H_. These results indicate that the independent effects of single factors on W_S_ and W_H_ exhibit a similar trend and that there is an interaction between different factors. To achieve optimal processing quality, the cutting speed should be increased, and the laser power should be decreased appropriately.

Range analysis quantifies how significantly factors influence the evaluation index by calculating the range of the evaluation index across different levels, thereby identifying dominant parameters. The cutting speed and laser power corresponding to levels 1 to 5 are listed in Table 2. As shown in Table 8, the mean value of W_S_ decreases from 53.34 μm to 23.00 μm with increasing cutting speed and increases from 25.44 μm to 44.87 μm with rising laser power. When considering W_S_ as the only quality metric, the optimal combination was achieved at level 5 of the cutting speed (100 mm/s) and level 1 of the laser power (1.15 W). The greater range value for cutting speed (R1 = 30.34) compared to laser power (R2 = 19.43) confirmed its more pronounced influence on W_S_. Similarly, W_H_ values decreased from 61.85 μm to 30.22 μm with higher cutting speeds and increased from 33.94 μm to 53.32 μm with higher laser power. When W_H_ is considered as the only evaluation index, as shown in Table 9, the ideal parameter combination is still 100 mm/s and 1.15 W. Comparing range values between two parameters (R1 = 31.63, R2 = 19.47) further demonstrated the dominant effect of the cutting speed on W_H_.

**Figure 11:**
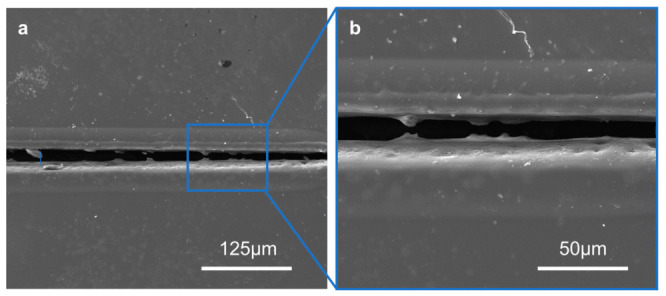
(**a**) Low-magnification SEM images and (**b**) high-magnification SEM images of the PA film under the optimal heat generation parameters.

With the repetition number of cutting fixed at one, the orthogonal experiment identified an optimal parameter combination for single cutting (Table 5, NO. 9). Under this process condition (80 mm/s, 1.67 W), the cut depth was less than 100 μm, so the repetition number of cutting was gradually increased to adjust the cutting depth and investigate its effect on the cutting quality. When the repetition number of cutting was increased to three, the PA film was completely cut through, as shown in Figure 11. Under this parameter combination, the width of the kerf is 23.6 ± 2.7 μm, and the width of HAZ is 28.4 ± 3.3 μm. SEM images of typical samples showed that the morphology of the kerf is ideal, with no apparent defects.

## 4. Conclusions

Firstly, based on systematic analysis of solid and gas byproducts, the UV picosecond laser cutting mechanism of the PA film was elucidated. Secondly, an orthogonal experiment was employed to investigate the influence of laser processing parameters on cutting quality and identify the optimal parameter combination. The principal conclusions derived from this study are as follows:

(1) Material removal during UV picosecond laser cutting of PA films is considered to involve both photochemical and photothermal effects. The high photon energy of the 355 nm laser may induce direct bond scission, while localized temperature rise caused by rapid energy deposition contributes to melting, vaporization, and heat diffusion, leading to the formation of the HAZ. The analyses of solid residues and thermal decomposition products provide supporting information for understanding possible material transformation pathways, although the exact chemistry occurring during laser ablation requires further investigation.
(2) No significant solid byproducts were observed during the laser–PA interaction. Meanwhile, the gas emissions exhibited a complex chemical composition containing potentially hazardous components. Therefore, adequate protection and ventilation measures should be implemented to prevent operators from inhaling or being exposed to harmful gases for extended periods.
(3) Increasing cutting speed and decreasing laser power effectively minimize the width of kerf and HAZ. However, excessive speed and insufficient power adversely affect material removal efficiency, with a diminishing rate of width reduction. Given these considerations, the optimal combination of process parameters within the investigated parameter ranges and the adopted two-stage experimental design framework was determined to be 80 mm/s, 1.67 W, and three times, under which a narrower kerf (23.6 ± 2.7 μm) and HAZ (28.4 ± 3.3 μm) were obtained, with and ideal kerf morphology achieved while maintaining sufficient cutting efficiency. Although the kerf width and HAZ width were used as the primary indicators of cutting quality in this study, additional characteristics such as edge roughness, edge uniformity, material redeposition, and debris formation should be systematically investigated in future work to provide a more comprehensive evaluation of UV picosecond laser cutting performance.

## Figures and Tables

**Table 1:** Main process parameters for the laser cutting system.

Factory	Value
Wavelength	355 nm
Pulse width	<10 ps
Cutting speed	0.1~10 m/s
Power	0~30 W
Focal length	167 mm
Beam quality parameter M2	1.2

**Table 2:** Factors and levels for the L25(52) orthogonal experiment.

Factory	Unit	Level				
		1	2	3	4	5
Cutting speed	mm/s	20	40	60	80	100
Power	W	1.15	1.67	2.14	2.66	3.18

**Table 3:** The atomic percentage at different positions around the kerf.

Atomic Percentage	Point A	Point B	Point C
C	84.93	87.13	86.20
O	15.07	12.87	13.80

**Table 4:** Composition table of the pyrolytic overflow gases.

Peak	RT	Area %	Library	CAS No.	2D	3D
1	1.492	4.18	Propane	000074-98-6	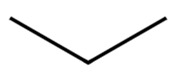	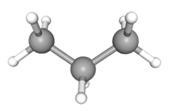
2	1.557	5.95	1,3-Butadiene	000106-99-0	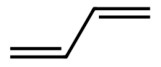	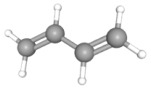
3	2.291	1.83	Tetrahydrofuran	000109-99-9	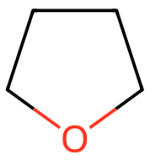	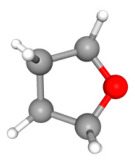
4	2.362	3.85	3-Buten-1-ol	000627-27-0	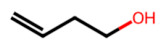	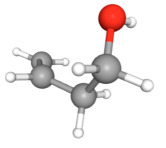
5	4.106	7.49	Cyclopentanone	000120-92-3	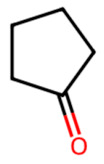	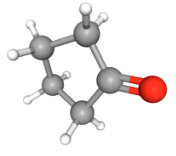
6	7.547	1.29	1,4-Butanediol	000110-63-4	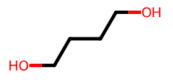	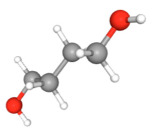
7	15.218	5.82	N-Methylmaleimide	000930-88-1	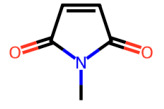	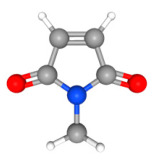
8	15.494	12.18	N-Methylmaleimide	000930-88-1	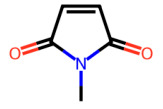	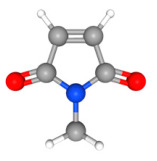
9	17.304	7.06	Adipic acid, dicyclobutyl ester	1000324-28-4	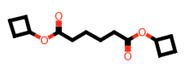	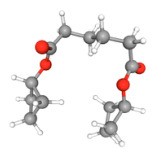

**Table 5:** L25(52) orthogonal experiments.

NO.	Power (W)	Cutting Speed (mm/s)	WS (μm)	WH (μm)	Cut Through
1	1.15	20	34.10	45.70	No
2	1.15	40	30.99	39.59	No
3	1.15	60	27.07	32.61	No
4	1.15	80	17.86	26.75	No
5	1.15	100	17.17	25.08	No
6	1.67	20	38.38	50.61	Yes
7	1.67	40	34.73	41.43	No
8	1.67	60	27.99	35.42	No
9	1.67	80	19.66	28.20	No
10	1.67	100	18.82	26.35	No
11	2.14	20	54.45	59.90	Yes
12	2.14	40	40.86	47.03	No
13	2.14	60	31.09	39.54	No
14	2.14	80	24.78	31.10	No
15	2.14	100	23.01	29.73	No
16	2.66	20	64.78	72.00	Yes
17	2.66	40	46.73	54.42	No
18	2.66	60	33.65	44.50	No
19	2.66	80	27.06	34.22	No
20	2.66	100	26.21	33.62	No
21	3.18	20	74.98	81.05	Yes
22	3.18	40	52.33	61.71	Yes
23	3.18	60	37.02	49.61	Yes
24	3.18	80	30.23	38.40	Yes
25	3.18	100	29.78	36.32	No

**Table 6:** ANOVA test for W_S_.

Factor	DOF	SS	MS	F	*p*	Contribution Effect %
Speed	4	9792.194	2448.049	1553.116	<0.001	64.44
Power	4	3899.763	974.941	618.532	<0.001	25.66
Speed × Power *	16	1425.461	89.091	56.522	<0.001	9.38
Error	50	78.811	1.576	-	-	0.52
Total	74	15,196.229	-	-	-	-

* Speed × Power represents the interaction effect between cutting speed and laser power.

**Table 7:** ANOVA test for W_H_.

Factor	DOF	SS	MS	F	*p*	Contribution Effect %
Speed	4	10,289.039	2572.260	1442.891	<0.001	68.20
Power	4	3872.575	968.144	543.073	<0.001	25.67
Speed × Power	16	836.566	52.285	29.329	<0.001	5.54
Error	50	89.136	1.783	-	-	0.59
Total	74	15,087.316	-	-	-	-

**Table 8:** Range analysis table based on W_S_.

	Cutting Speed (mm/s)	Power (W)
$\bar{K}$1	53.34	25.44
$\bar{K}$2	41.13	27.92
$\bar{K}$3	31.36	34.84
$\bar{K}$4	23.92	39.69
$\bar{K}$5	23.00	44.87
Optimal level	5	1
Rj	30.34	19.43
Order of range	R1 > R2	-

**Table 9:** Range analysis table based on W_H_.

	Cutting Speed (mm/s)	Power (W)
$\bar{K}$1	61.85	33.94
$\bar{K}$2	48.84	36.40
$\bar{K}$3	40.33	41.46
$\bar{K}$4	31.73	47.75
$\bar{K}$5	30.22	53.42
Optimal level	5	1
Rj	31.63	19.47
Order of range	R1 > R2	-

## Data Availability

The datasets generated during and/or analyzed during the current study are available from the corresponding author on reasonable request.
